# Pneumolysin suppresses the initial macrophage pro‐inflammatory response to *Streptococcus pneumoniae*

**DOI:** 10.1111/imm.13546

**Published:** 2022-07-28

**Authors:** Jimstan Periselneris, Carolin T. Turner, Giuseppe Ercoli, Gabriella Szylar, Caroline M. Weight, Teresa Thurston, Matthew Whelan, Gillian Tomlinson, Mahdad Noursadeghi, Jeremy Brown

**Affiliations:** ^1^ Centre for Inflammation and Tissue Repair, Division of Medicine University College Medical School London UK; ^2^ Division of Infection and Immunity University College London London UK; ^3^ MRC Centre for Molecular Bacteriology and Infection Imperial College London London UK

**Keywords:** epithelial immunity, inflammation, pneumolysin, *Streptococcus pneumoniae*

## Abstract

Published data for the *Streptococcus pneumoniae* virulence factor Pneumolysin (Ply) show contradictory effects on the host inflammatory response to infection. Ply has been shown to activate the inflammasome, but also can bind to MRC‐1 resulting in suppression of dendritic cell inflammatory responses. We have used an in vitro infection model of human monocyte‐derived macrophages (MDM), and a mouse model of pneumonia to clarify whether pro‐ or anti‐inflammatory effects dominate the effects of Ply on the initial macrophage inflammatory response to *S. pneumoniae*, and the consequences during early lung infection. We found that infection with *S. pneumoniae* expressing Ply suppressed tumour necrosis factor (TNF) and interleukin‐6 production by MDMs compared to cells infected with *ply*‐deficient *S. pneumoniae*. This effect was independent of bacterial effects on cell death. Transcriptional analysis demonstrated *S. pneumoniae* expressing Ply caused a qualitatively similar but quantitatively lower MDM transcriptional response to *S. pneumoniae* compared to *ply*‐deficient *S. pneumoniae*, with reduced expression of TNF and type I IFN inducible genes. Reduction of the MDM inflammatory response was prevented by inhibition of SOCS1. In the early lung infection mouse model, the TNF response to *ply*‐deficient *S. pneumoniae* was enhanced and bacterial clearance increased compared to infection with wild‐type *S. pneumoniae*. Overall, these data show Ply inhibits the initial macrophage inflammatory response to *S. pneumoniae*, probably mediated through SOCS1, and this was associated with improved immune evasion during early lung infection.

## Graphical abstract

The *Streptococcus pneumoniae* cholesterol binding toxin pneumolysin has previously been shown to have both pro‐ and anti‐inflammatory effects. Here, we show that the anti‐inflammatory effects dominate during the early macrophage response to *S. pneumoniae*. Extracellular pneumolysin inhibits the pro‐inflammatory transcriptional response to *S. pneumoniae* via SOCS‐1‐mediated inhibition of NFκB translocation. This results in reduced release of TNF by macrophages, and improved immune evasion during the initial interactions of *S. pneumoniae* with the host in a mouse model of pneumonia.

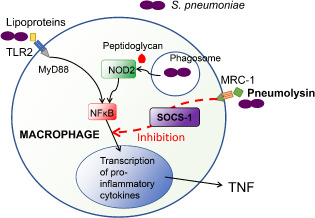

## INTRODUCTION

*Streptococcus pneumoniae* is a common nasopharyngeal commensal that is also a significant bacterial pathogen causing infections such as pneumonia, meningitis, and septic shock [1]. Macrophages are essential for the rapid recognition and phagocytosis of *S. pneumoniae* as well as mounting the initial inflammatory response to infection and subsequent recruitment of other immune effectors [2, 3, 4]. Macrophage recognition of *S. pneumoniae* by pro‐inflammatory signalling molecules such as toll‐like receptors (TLRs) results in translocation of nuclear factor‐κB (NFκB) into the nucelus, release of tumour necrosis factor (TNF) and transcription of other pro‐inflammatory cytokines such as interleukin 6 (IL‐6) [5, 6, 7].

Pneumolysin (Ply), a 53 kDa pore‐forming *S. pneumoniae* toxin, is essential for pathogenesis in animal models of infection [8, 9, 10]. Ply binds to cholesterol containing membranes, then polymerizes to create pores in the host cell membrane. At high concentrations Ply is toxic to host cells, inducing cell death by different pathways including apoptosis [4, 11] and necroptosis [12]. At sub‐lytic concentrations Ply has additional pleiotropic effects on host cells that are often pro‐inflammatory. These may be mediated by interaction with TLR4 [13, 14], or by facilitating leak of bacterial DNA and peptidoglycan from phagosomes to activate cytosolic sensors [15, 16], and activation of the inflammasome [13]. In animal models, Ply induces leukocyte infiltrates and protein leak into bronchoalveolar lavage fluid (BALF) and largely replicates the histological appearances of pneumonic lung inflammation [17, 18, 19]. Hence, Ply is generally considered a pro‐inflammatory molecule that contributes to the pathological damage caused by *S. pneumoniae* infections. However, recent data suggest Ply has anti‐inflammatory effects. Ply‐dependent killing of dendritic cells (DCs) and of cardiac macrophages reduces inflammatory cytokine secretion [20, 21]. In addition, recently Ply recognition by the cell surface receptor mannose receptor 1 (MRC‐1) has been described, and this causes increased expression of suppressor of cytokine responses 1 (SOCS1), and suppressed inflammatory responses to *S. pneumoniae* [22]. siRNA knockdown of MRC‐1 or treatment with an anti‐MRC‐1 antibody increased the TNF response to Ply‐expressing *S. pneumoniae* from DCs and ex vivo alveolar macrophages, as well as in a mouse model of pneumonia [22].

Overall, there are contradictory data on how Ply affects the macrophage inflammatory response to *S. pneumoniae*, a key interaction for the pathogenesis of infection. To address this we have used in vitro human and in vivo mouse infection models to define in detail the effects of Ply on the initial macrophage‐dependent inflammatory responses to *S. pneumoniae*.

## MATERIALS AND METHODS

### Bacterial strains and culture

Pneumolysin‐deficient Δ*ply* serotype 4 TIGR4, and serotype 3 and 23F *S. pneumoniae* strains were constructed by complete deletion of the ply gene. The clinical isolates INV104B, 03.3038, and NCTC7465 were kind gifts from Professor T Mitchell. Serotype 2 (D39), its isogenic Ply‐deficient mutant and the complemented D39 strain were kind gifts from Professor J Weiser. *S. pneumoniae* were labelled for microscopy experiments using 6‐carboxyfluorescein succinimidyl ester (Molecular Probes) as previously described [23]. The red cell lysis assays to assess Ply function were performed using 2% horse blood and supernatant absorbance at 540 nm as previously described [24].

### Cell culture experiments

Peripheral blood mononuclear cells from healthy volunteers were differentiated into monocyte‐derived macrophages (MDM) as previously described [25]. Experiments using MDMs were approved by the joint University College London/University College Hospitals National Health Service Trust Human Research Ethics Committee (Ref: 3076/001). For infection experiments MDMs were cultured in RPMI with 5% pooled human AB serum (Sigma Aldrich). To inhibit SOCS1 10 μM of inhibitor peptide (iKIR—DTHFRTFRSHADYRRI) or scrambled control peptide (DTHFARTFARSHADYRRI) (Genscript) were added 1 hr before incubation with bacteria [26]. MDM supernatant ELISAs were carried out using R&D Systems (Abingdon) Duo kits as previously described [27]. For the galectin 3 phagosome assay THP1 cells expressing mCherry Galectin 3 were differentiated with 25 ng/ml PMA then 200 μg/ml gentamicin was added. 1:1 trypan blue solution was added to quench extracellular bacteria fluorescence, before fixing in 4% paraformaldehyde, mounting onto microscope slides and images captured using Zeiss LSM510 confocal microscope. The number of cells, bacteria and galectin 3 positive bacteria were recorded from at least 25 cells. At least three images were taken of each strain per experiment. Zen software was used for colocalisation analysis. The antibiotic protection assay was performed after MDM were infected with bacteria, then the addition of gentamicin (200 μg/ml) to kill extracellular bacteria to some wells, then saponin to lyse the cells to assess internalized bacterial numbers. The microscopy NFκB translocation assays was performed as previously described [28].

### Cell viability and death assays

MDM viability was assessed by measuring supernatant lactate dehydrogenase levels (CyQUANT LDH cytotoxicity assay; C20300; Thermo Fisher Scientific) and the Cell Counting Kit‐8 reagent (Sigma; 96992) using absorbance at 460 nm to quantify formazan dye levels (directly proportional to the number of living cells). To assess cell death using microscopy MDMs were stained with Live Cell imaging solution (Invitrogen) containing 5 μM Hoechst33342 (Thermo Fisher Scientific) nuclear marker and 1 μM propidium iodide (PI; nuclear staining identifies dead cells). Images for the cell death and NFκB translocation assays were acquired using the WiScan Hermes High‐Content Imaging System and automated image analysis performed using the ‘Quantitative Cytometry’ module of the Athena Image analysis software (IDEA Bio‐Medical).

### RNA microarray whole genome transcription and bioinformatic analyses

MDM RNA extraction was performed with Quiagen RNeasy Mini kit (Quiagen). cDNA was synthesized with qScript cDNA SuperMix (QuantaBiosciences). qPCR was performed using TaqMan gene expression assays on a Realplex Mastercycler (Eppendorf) with GAPDH as housekeeping gene. Normalization for RNA quality, transcriptional profiling by Agilent microarrays and subsequent data processing were performed as previously described [29, 30, 31]. Microarray data are available in ArrayExpress (https://www.ebi.ac.uk/arrayexpress/, accession number: E‐MTAB‐8947). Principal component analysis was performed with the prcomp function in R (version 3.6.0). Statistically significant genes upregulated were defined using paired *t* tests in Multi Experiment Viewer v4.9 (http://www.tm4.org/mev.html). Ingenuity pathway analysis (IPA) (Qiagen) was used to identify the interactome of differentially expressed genes, and to probe directly interacting genes further for predicted upstream regulators. The 10 most significant upstream regulators with activation *z*‐scores >2 were visualized as networks in Gephi v0.9.2, and their statistical enrichment compared by hierarchical clustering and visualized in a heatmap (https://software.broadinstitute.org/morpheus). Reactome pathway enrichment of differentially expressed, interacting genes was analysed with the XGR R package [32]. For visualization purposes, 20 pathway groups were identified by hierarchical clustering, with the pathway with the largest total number of genes selected to provide a representative annotation. Gene signatures for type 1 IFN‐ or TNF‐inducible macrophage responses and LPS‐inducible genes were previously published [30, 33, 34]. Gene module scores were calculated as mean expression of the constituent gene names in each module. TaqMan gene expression assays were run on a Realplex Mastercycler (Eppendorf), Cycle threshold (*C*_t_) determined and analysed as Δ*C*_t_ for relative expression values using GAPDH as a housekeeping gene [29].

### Mouse infection model

Murine work was performed within Home Office guidelines (project licence PPL70/7361). Female CD1 5‐week‐old mice (Charles Rivers) were anaesthetised with isoflurane and inoculated intranasally with 5 × 10^6^ CFU in 50 μl of phosphate buffered saline as previously described [2, 7, 27]. Mice were euthanised with intraperitoneal pentobarbital, and BALF, and lungs (homogenized using cell strainers; Falcon) obtained to calculate bacterial CFU by plating serial dilutions and cytokine levels using commercial ELISAs (R&D Systems). BALF cell counts were obtained using a haemocytometer.

### Statistical analysis

Statistical analyses of data were performed with GraphPad Prism V 9. Data were analysed by unpaired *t*‐test when comparing two groups, and one‐ or two‐way ANOVA with Tukey's or Sidak's multiple comparisons test respectively for multiple groups. Non‐parametric data (e.g., CFU) were analysed by Mann–Whitney *U* test when comparing two groups, and Kruskal–Wallis with Dunn's multiple comparisons test for multiple groups.

## RESULTS

### Increased MDM inflammatory cytok*ine* responses in response to infection with TIGR4Δ*ply S. pneumoniae*

**FIGURE 1:**
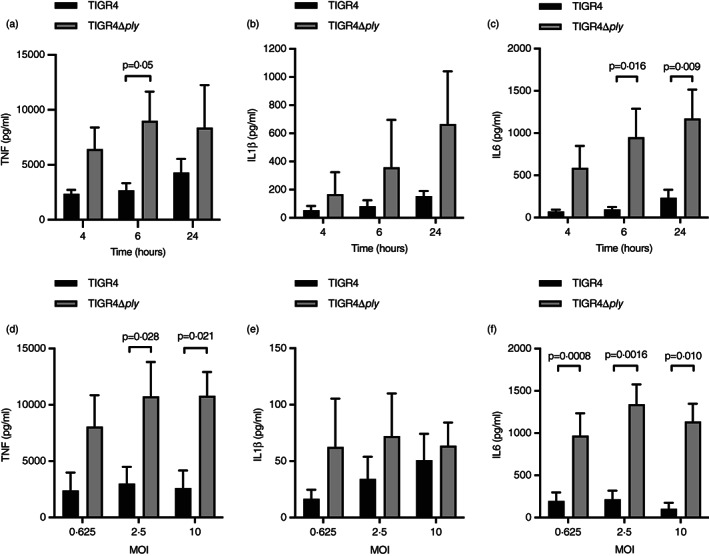
TIGR4Δ*ply* induces higher levels of pro‐inflammatory cytokine responses by MDMs than TIGR4. *S. pneumoniae* (TIGR4 in black and TIGR4Δ*ply* in grey) were incubated with MDMs and the supernatant analysed using ELISAs for TNF (a and d), IL‐1β (b and e), and IL‐6 (c and f) levels (pg/ml) for different timepoints at an MOI 10 (a–c) or various MOI after 6‐h incubation (d–f). Data are presented as means ± SEM of at least three experiments, and analysed by two‐way ANOVA with Tukey's multiple comparisons test.

**FIGURE 2:**
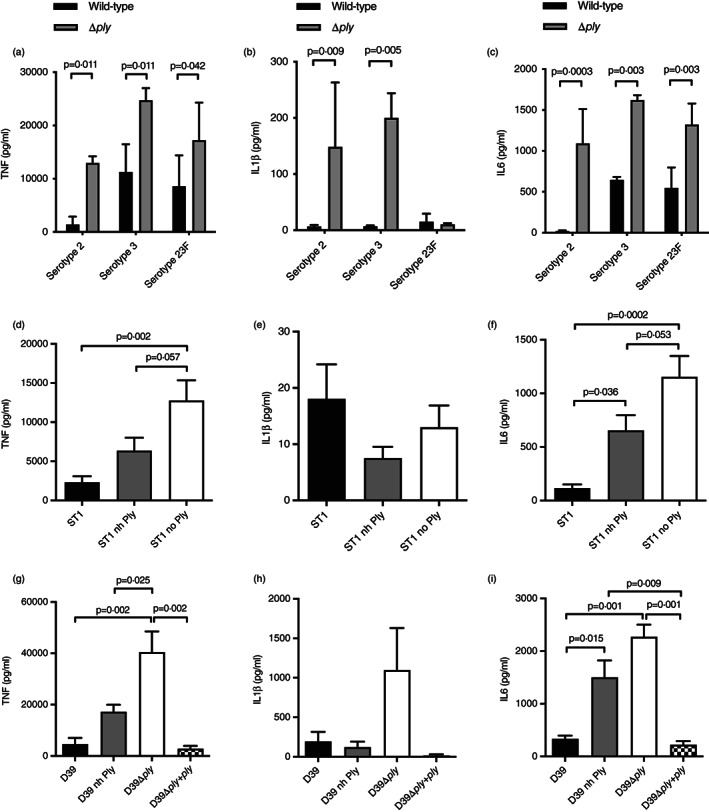
Ply suppresses MDM inflammatory responses for multiple *Streptococcus pneumoniae* strains. *S. pneumoniae* strains were incubated with MDMs at an MOI 10 and the supernatant analysed after 6 h using ELISAs for TNF (a, d, and g), IL‐1β (b, e, and h), and IL‐6 (c, f, and i) levels (pg/ml). (a–c) Results for MDMs incubated with wild‐type (black) and isogenic Δ*ply* mutants (grey) of serotype 2, 3 and 23F strains. (d–f) Results for MDMs incubated with serotype 1 clinical isolates that express wild‐type ply (black), non‐haemolytic ply (grey), or no ply (white). (g–i) Results for MDMs incubated with D39 (serotype 2) and D39 isogenic mutant strains expressing non‐haemolytic or no ply, and a complemented strain. Data for all panels are presented as means ± SEM of three experiments and analysed by two‐way ANOVA with Sidak's multiple comparisons test.

The effect of Ply on macrophage inflammatory responses was evaluated using an established MDM *S. pneumoniae* infection model [7, 27] and the TIGR4 and TIGR4Δ*ply* strains. The red cell lysis assay confirmed only the TIGR4 strain had Ply activity (Figure S1A). Compared to infection with TIGR4Δ*ply*, MDMs infected with wild‐type TIGR4 produced lower levels of TNF (at 6 h) and IL‐6 (6 and 24 hr) across multiplicities of infection ranging from 0.625 to 10 (Figure 1a–f). Although there was a trend for increased IL‐1β production from MDMs infected with the TIGR4Δ*ply* strain, these differences were not statistically significant. To confirm Ply‐dependent suppression of MDM pro‐inflammatory responses was not strain‐specific, the experiments were repeated with serotype 2, 3, and 23F *S. pneumoniae* strains. There were reduced levels of TNF, IL‐1β (for serotype 2 and 3 strains) and IL‐6 in MDM supernatants infected with wild‐type compared to Ply‐deficient strains (Figure 2a–c). This pattern was recapitulated after MDMs were infected with serotype 1 *S. pneumoniae* clinical isolates that do not express Ply or (to a less marked extent) express a non‐haemolytic version of Ply [24, 35] (Figure 2d–f). Complementation of the D39 *S. pneumoniae ply*‐deficient strain with *ply* alleles encoding a non‐haemolytic or a fully haemolytic Ply (Figure 1B) also impaired the MDM cytokine response, again with a reduced effect for the non‐haemolytic Ply strain [36] (Figure 2g–i). These data show that Ply‐mediated suppression of MDM inflammatory responses occurs with multiple *S. pneumoniae* strains, and the effect was partially dependent on the pore‐forming ability of Ply.

### Differences in bacterial numbers, phagocytosis, or cell death do not explain the reduced MDM inflammatory responses to TIGR4

**FIGURE 3:**
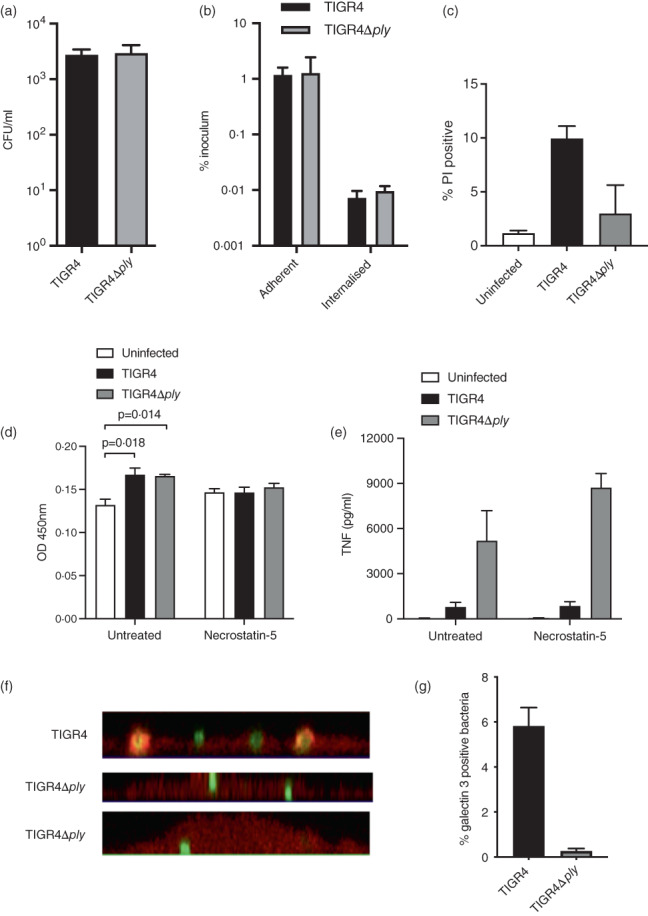
Effects of ply on *Streptococcus pneumoniae* growth, phagocytosis, cell death, and phagosome integrity in the MDM infection model. (a and b) *S. pneumoniae* growth in the presence of and uptake by macrophages is unaffected by ply. MDM were incubated with TIGR4 (black) or TIGR4Δ*ply* (grey) at MOI 10 and supernatant cultured after 6 h plated to determine (a) bacterial CFU in the supernanant, and (b) determine adherent and internalized bacteria as a percentage of the inoculum using an antibiotic protection assay. (c) MDM were incubated with TIGR4 or TIGR4Δ*ply* at MOI 10 for 6 h, then stained with propidium iodide as a marker of loss of nuclear integrity. The proportion of MDM that stained positive were measured using Hermes high‐content imaging system (magnification ×10) and automated image analysis. Data are shown as means ± SEM of three experiments and analysed by one‐way ANOVA with Tukey's multiple comparisons test. (d and e) Effects of inhibition of necroptosis; 100 μM Necrostain (N0164, sigma) was added for 1 h to MDM before infection with TIGR4 or TIGR4Δ*ply* (MOI 10) for 6 h. (d) Cell viability was assessed by measuring supernatant LDH levels, and (e) supernatant TNF levels measured using ELISAs. Data from four experiments are presented means ± SEM and analysed by two‐way ANOVA with Sidak multiple comparisons test. (f and g) Confocal microscopy for localisation of FAM‐SE labelled TIGR4 and TIGR4Δ*ply* (green) after incubation with THP‐1 cells expressing mCherry galectin 3 (red, MOI 100). Images were acquired after quenching fluorescence from external bacteria with trypan blue. The proportion of galectin 3 positive bacteria were measured by microscopy. Sample images are shown in panel F demonstrating that TIGR4 bacteria colocalise with galactin 3, whereas TIGR4Δ*ply* do not. The proportion of galectin 3 positive bacteria for TIGR4 and TIGR4Δ*ply* are shown in panel (g) as means ± SEM from four experiments.

Under the experimental conditions used during the MDM infection experiments there were no differences in bacterial survival and internalization by MDMs between TIGR4Δ*ply* and TIGR4 (Figure 3a,b). As previous data has shown Ply‐induced cell death reduced DC and cardiac macrophages inflammatory responses [12, 21], we quantified cell death in our MDM infection model using three separate assays; PI staining (a marker of loss of nuclear membrane integrity), supernatant LDH levels, and formazan dye levels (proportional to cell metabolic activity). All three assays showed that under our experimental conditions between approximately 70%–90% of MDMs remained viable (Figures 3c and S2). Although cell death was sometimes slightly higher for MDMs incubated with wild‐type TIGR4 compared to TIGR4Δ*ply* these differences were inconsistent and statistically not significant. The small degree of MDM cell death after *S. pneumoniae* infection was reversed by inhibition of necroptosis (Figure 3d), but not apoptosis (Figure S2B). Inhibition of necroptosis or apoptosis did not reverse attenuation of TNF production by wild‐type bacteria expressing Ply (Figures 3e and 2C,D). Hence increased cell death in response to Ply was unlikely to be the main cause of differences in MDM TNF responses between Ply+ and Ply− strains. Confocal microscopy demonstrated a higher proportion of MDM phagosomes containing bacteria were associated with galectin 3, a marker for vacuole integrity, after infection with TIGR4 compared to infection with TIGR4Δ*ply* (Figure 3f,g). This result indicated that Ply created functional pores in the phagosomal membrane, which potentially could affect the MDM inflammatory response.

### Transcriptional profiling demonstrated Ply suppressed the MDM innate immune response to external bacteria

**FIGURE 4:**
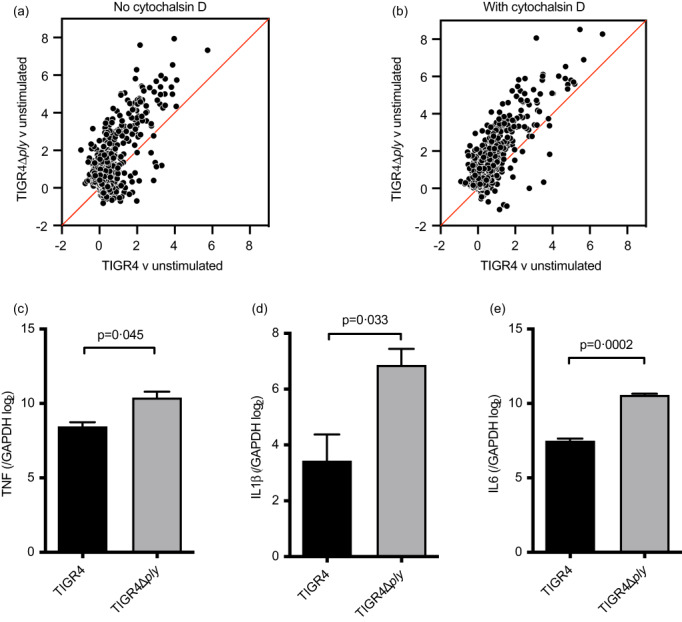
Transcriptome analysis of MDMs after incubation with *Streptococcus pneumoniae* TIGR4, TIGR4Δ*ply*, (MOI 10) or controls for 4 h analysed using microarrays. (a and b) The log2 fold difference of genes upregulated by either TIGR4 or TIGR4Δ*ply* in the absence (a) or presence (b) of cytochalasin D is shown in scatter plots. The red line indicates the hypothetical line of perfect correlation. (c–e) Taqman qPCR measurement of MDM gene expression for individual pro‐inflammatory cytokines after 4‐h incubation with TIGR4 or TIGR4Δ*ply* at MOI 10. Results from three donors are presented as change in cycle threshold with GAPDH as a housekeeping gene for TNF (c), IL‐1β (d), and IL‐6 (e), and analysed by analysed by paired *t* tests.

Genome‐wide transcriptional profiling provided a comprehensive overview of Ply effects on MDM responses to *S. pneumoniae*. We have previously shown MDM responses to TIGR4 were largely mediated by external bacteria [27], yet the galectin 3 data suggested leak of pro‐inflammatory phagosome contents into MDM cytosol will be limited to MDMs containing intracellular wild‐type *S. pneumoniae*. Hence, expression of significantly upregulated genes were compared between each strain and unstimulated MDMs in the presence and absence of cytochalasin D which blocks macrophage phagocytosis of *S. pneumoniae* [27] (Figure S3A,B). In general, quantitative gene expression changes among the integrated list of upregulated transcripts following bacterial stimulation were greater in response to the TIGR4Δ*ply* strain compared to TIGR4 (Figure 4a) with or without cytochalasin D (Figure 4b). The list of genes that showed greater upregulation by TIGR4Δ*ply* included the canonical pro‐inflammatory genes for TNF, IL‐6, IL‐1β, and was confirmed by qPCR (Figure 4c–e). Interestingly, upregulated genes were not limited to pro‐inflammatory responses and included the anti‐inflammatory gene IL‐10 (Table 1) although this was not associated with differences in MDM supernantant IL‐10 levels (data not shown).

**TABLE 1:** MDM transcriptional responses (mean expression measured using microarrays) of selected genes after infection with TIGR4 compared to TIGR4Δ*ply*

Gene	Uninfected	SD	TIGR4	SD	TIGR4Δ*ply*	SD	Statistically different
TNF	9.485	1.18	15.233	0.34	16.811	0.56	*p* = 0.020
IL‐6	5.946	0.33	9.920	0.54	13.890	0.19	*p* < 0.0001
IL‐1β	11.587	1.57	14.837	0.54	16.556	0.52	*p* = 0.010
IL‐10	6.857	0.21	7.786	0.58	10.953	1.16	*p* < 0.0001
MRC‐1	10.226	0.61	9.547	0.70	9.816	0.38	Non sig
SOCS1	7.446	0.45	10.201	0.37	10.858	0.62	Non sig

*Note*: Assessed by two‐way ANOVA and Tukey's multiple comparisons test.

### Systems level analysis of ply‐mediated effects on MDM innate immune responses

**FIGURE 5:**
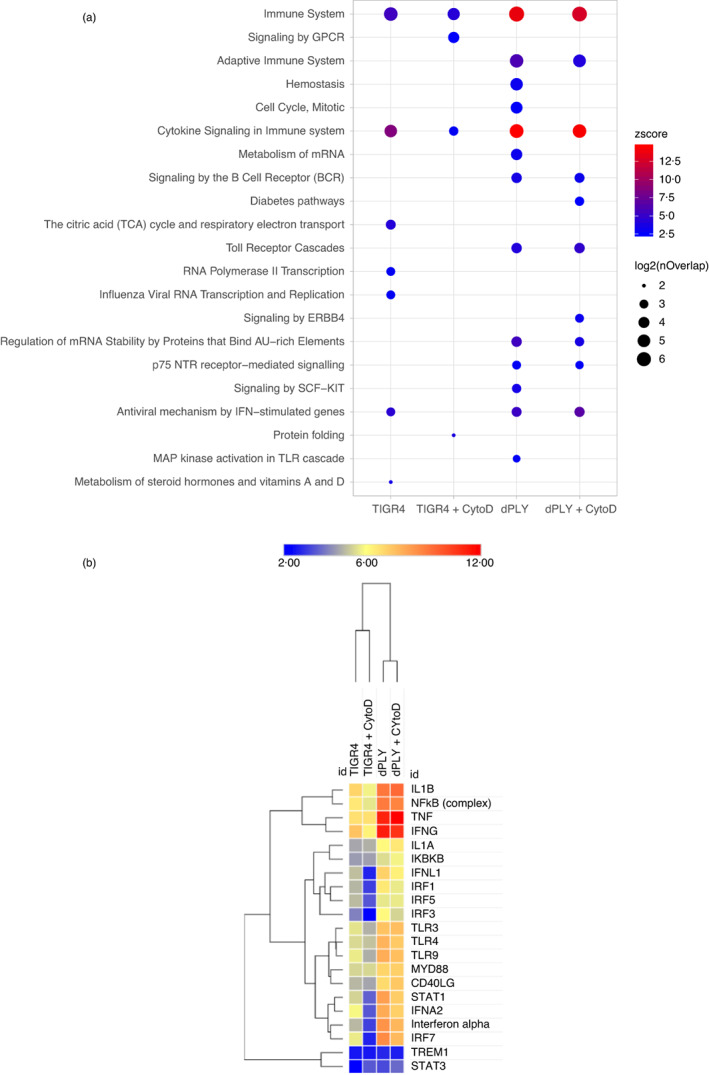
Bioinformatic pathway analysis downstream of the MDM transcriptome response measured using microarrays after 4‐h incubation with *Streptococcus pneumoniae* (MOI 10). (a) Statistical enrichment (*Z* score) of Reactome pathways associated with upregulated transcripts in MDM incubated with TIGR4 or TIGR4Δ*ply* in the presence or absence of cytochalasin D. The size of the nodes represents the number of genes activated in that condition for the named pathway. The colour of the nodes represent the statistical (*Z* score) enrichment of these pathways in each condition. These data were obtained from three experiments with separate donors. (b) Heat map depicting statistical enrichment (activation *Z* score) of predicted upstream regulators for upregulated transcripts in MDMs incubated with TIGR4 or TIGR4Δ*ply* in the presence or absence of cytochalasin D, clustered by Euclidean distance for experimental condition (columns) and upstream regulators (rows).

**FIGURE 6:**
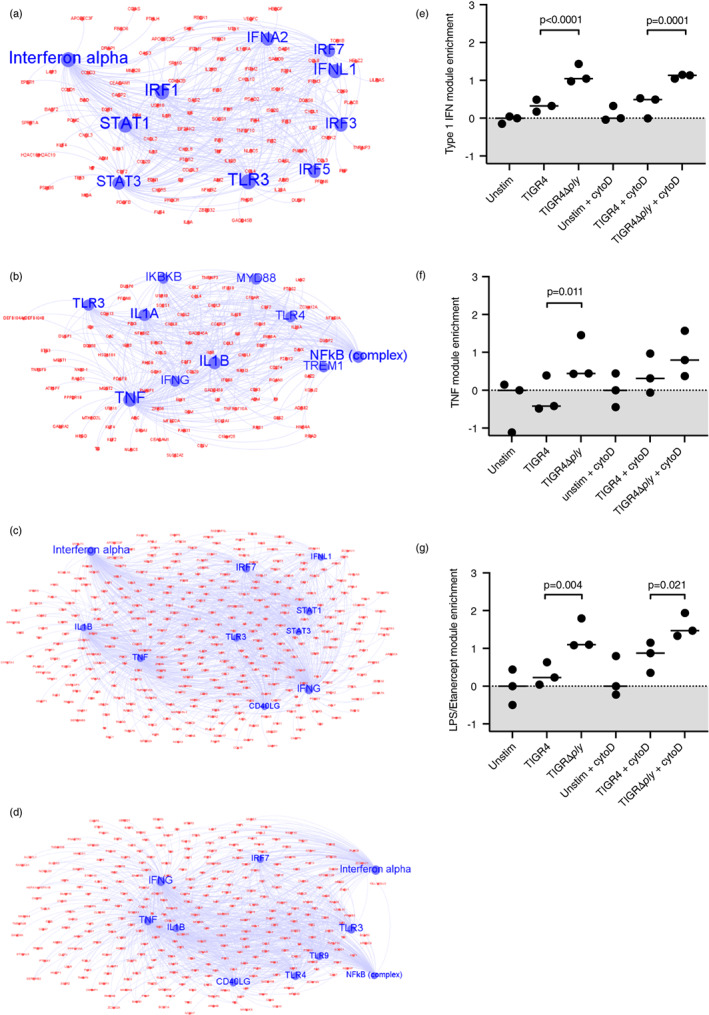
Pathway analysis of transcriptome data for MDMs after incubation with *Streptococcus pneumoniae* TIGR4, TIGR4Δ*ply*, (MOI 10) or controls for 4 h. (a–d) Identification of the top 10 predicted upstream regulators (blue nodes) of upregulated transcripts (red nodes) in MDMs stimulated with TIGR4 or TIGR4Δ*ply* with or without cytochalasin D using ingenuity pathway analysis. (a) TIGR4, (b) TIGR4 with cytochalasin D, (c) TIGR4Δ*ply*, and (d) TIGR4Δ*ply* with cytochalasin D. (e–h) Pre‐defined and validated gene expression signatures [30, 33, 34] were used to analyse enrichment for specific modules in the transcriptome data; (e) type I interferon, (f) TNF, (g) LPS with etanercept, representing endogenous TNF production. Data were analysed by paired *t* tests.

To compare MDM responses to TIGR4 and TIGR4Δ*ply* strains at systems level, upregulated transcripts in the presence and absence of cytochalasin D were limited to gene products predicted to interact using the interactome database in IPA. Bioinformatic analyses of each interactome was used to identify statistically enriched biological pathways and upstream molecules predicted drive their expression (Figures 5 and 6a–d). Enrichment of overlapping immune response pathways and upstream regulators was evident in each of the four experimental conditions (Figure 5a), but the statistical enrichment for the upstream regulators were higher in MDM infected with TIGR4Δ*ply* compared to TIGR4. In addition, TIGR4Δ*ply* invoked exaggerated responses of independently derived TNF and type 1 IFN inducible gene signatures compared to TIGR4 (Figure 6e–g). In this analysis, no effect of cytochalasin D was evident in the overall transcript levels in MDM infected with either strain. However, upstream regulator analysis suggested that cytochalasin D caused a lower statistical enrichment for responses predicted to be regulated by type 1 IFN responses (Figure 5b), suggesting that intracellular TIGR4 was associated with increased type 1 IFN pathway responses. Overall, the transcriptional analyses showed Ply broadly inhibited MDM innate immune responses to *S. pneumoniae*, with TIGR4Δ*ply* infection associated with quantitatively greater but qualitatively similar MDM pro‐inflammatory transcriptional responses compared to TIGR4. These differences were largely independent of bacterial internalization, reflecting the dominance of external *S. pneumoniae* for inducing the MDM inflammatory response [27].

### Ply‐mediated inhibition of MDM inflammatory to *S. pneumoniae* was associated with reduced NFκB activation and reversed by inhibition of SOCS1

**FIGURE 7:**
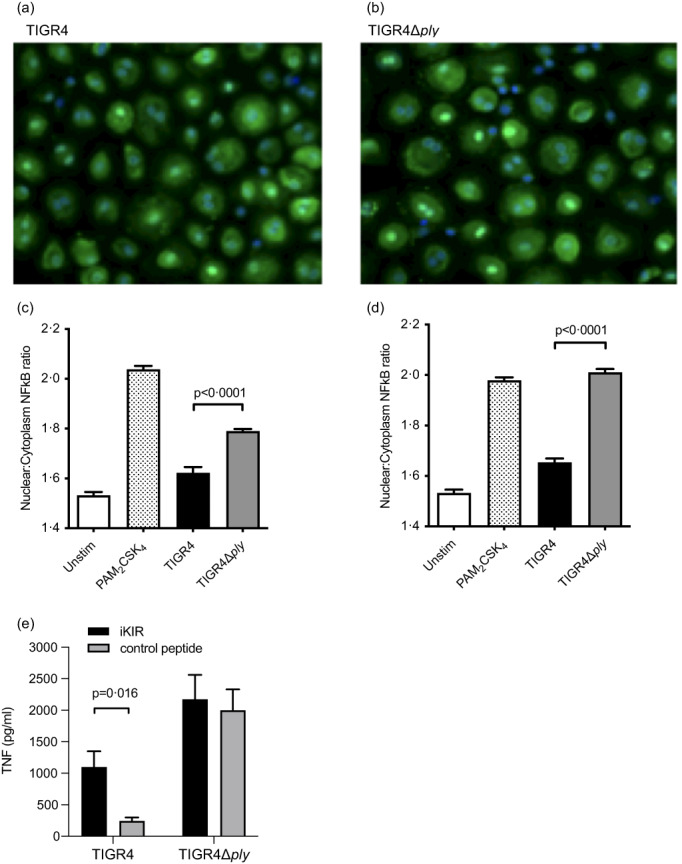
Ply effects on MDM NFκB nuclear translocation and SOCS1 mediated TNF responses. (a–d) Nuclear translocation of NFκB for MDMs infected with TIGR4 or TIGR4Δ*ply* (MOI 10) using PAM_2_CSK_4_ (TLR2 agonist). The nuclear to cytoplasm ratio of NFκB was measured using confocal microscopy at 1 h. Sample microscopy images after 1 h of infection with NFκB shown in green and nuclei shown in blue of (a) TIGR4 and (b) TIGR4Δ*ply*. (c and d) NFκB nuclear to cytoplasm ratio after (c) 1 h or (d) 2 h of infection. Data from three experiments presented as means ± SEM and analysed by unpaired t‐test. (E) MDM supernatant TNF responses measured using ELISA 6 h after incubation with *Streptococcus pneumoniae* strains (MOI 10) in the presence or absence of the peptide inhibitor (iKIR, 10 μM) of human SOCS1 or a scrambled control peptide inhibitor (10 μM). Data for panels are presented as means ± SEM and analysed by two‐way ANOVA with Sidak's multiple comparisons test.

To assess the effects of Ply on pro‐inflammatory signalling, nuclear translocation of NFκB RelA was assessed in MDM 1 and 2 hr after infection with *S. pneumoniae* TIGR4 or TIGR4Δ*ply*. NFκB translocation into nuclei was reduced in MDMs infected with TIGR4 compared to TIGR4Δ*ply* (Figure 7a–d), consistent with Ply‐mediated attenuation of pro‐inflammatory intracellular signalling pathways in response to *S. pneumoniae* infection. Ply inhibits DC inflammatory responses to *S. pneumoniae* by direct binding to the cell surface mannose receptor MRC‐1which then is thought to inhibit pro‐inflammatory signalling pathways through SOCS1 expression [22]. Our transcriptional data showed MRC‐1 and SOCS1 were expressed by MDMs (Table 1), and inhibition of SOCS1 using a specific peptide increased the MDM TNF response to TIGR4 but not to TIGR4Δ*ply* (Figure 7e). These results in combination with the transcriptome and MDM infection data support the hypothesis that Ply suppresses early MDM pro‐inflammatory cytokine responses to *S. pneumoniae* through SOCS1 mediated inhibition of NFκB translocation to the nucleus.

### Ply suppresses TNF responses in a murine model of early lung infection

**FIGURE 8:**
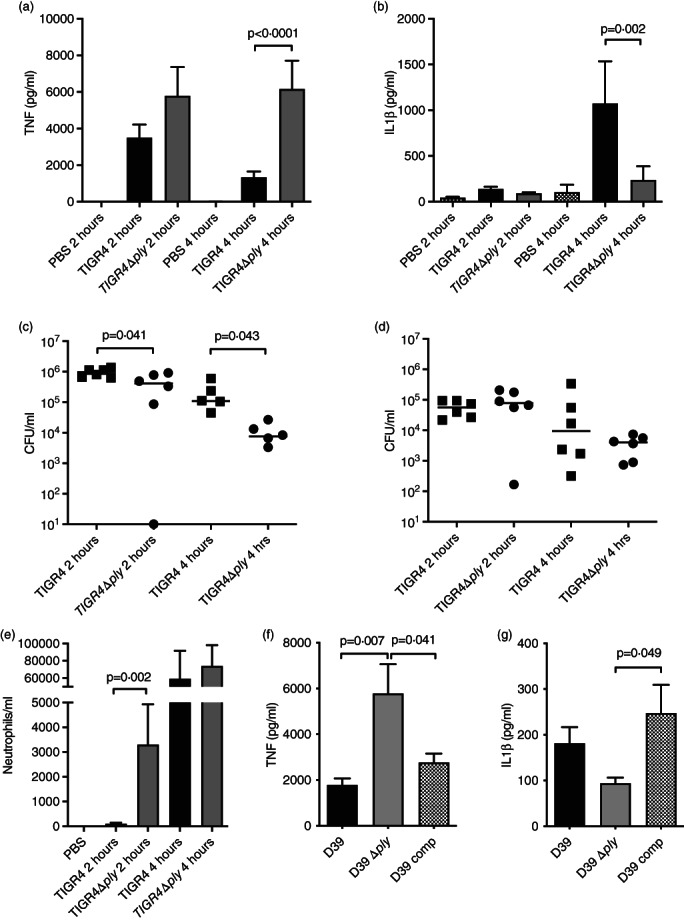
Effects of Ply in a mouse model of early lung infection. (a–e) Female CD1 mice (six in each group) were infected intranasally with 5 × 10^6^ CFU of *Streptococcus pneumoniae* TIGR4 or TIGR4Δ*ply* and BALF obtained 2 or 4 h after infection. (a and b) BALF (a) TNF or (b) IL‐1β levels measured by ELISA and shown as means ± SEM analysed by unpaired *t* tests. (c and d) Bacterial CFU 4 h post‐infection measured by plating serial dilutions recovered from (c) BALF or (d) lung homogenates. The data are displayed as individual data points, with bars representing medians, and analysed by Mann–Whitney *U* tests. (e) BALF neutrophil concentrations 2 and 4 h after infection presented as mean ± SEM and analysed by one‐way ANOVA with Tukey's multiple comparisons test. (f and g) BALF (f) TNF and (g) IL‐1β levels measured by ELISA 4 h after infection of female CD1 mice (six in each group) with *S. pneumoniae* D39, D39Δ*ply*, or the D39Δ*ply ply* complemented strain (D39 comp). Data are shown as means ± SEM analysed by two‐way ANOVA with Sidak multiple comparisons test.

The effects of Ply on macrophage inflammatory responses during infection was assessed using an *S. pneumoniae* mouse model of early lung infection. In this model TNF levels in BALF are largely dependent on alveolar macrophage responses [2, 27]. In line with the in vitro data, 4 hr after infection BALF TNF levels were higher in animals infected with TIGR4Δ*ply* compared to TIGR4 (Figure 8a). In contrast and compatible with the known effect of Ply on inflammasome activation [13], BALF IL‐1β levels were lower in mice infected with TIGR4Δ*ply* (Figure 8b). These differences in BALF cytokines were associated with around 1 log_10_ greater CFU in BALF 4 hr after infection for TIGR4 compared to TIGR4Δ*ply* (Figure 8c). There were no differences in lung CFU, which represent only about 1% of the bacterial load at this timepoint (Figure 8d). Furthermore, TIGR4Δ*ply* infection resulted in faster recruitment of neutrophils to BALF compared TIGR4 infection (Figure 8e). Repeating mouse infection experiments at 4 hr with the D39 *S. pneumoniae* strain demonstrated similar BALF cytokine results, with increased TNF and reduced IL‐1β levels for mice infected with D39Δ*ply* compared to D39 or the *ply* complemented strain (Figure 8f,g). Overall, these data confirm that during early lung infection Ply suppresses BALF pro‐inflammatory cytokine responses, reduces the rate of neutrophil recruitment to the lungs, and is associated with improved immune evasion by *S. pneumoniae*.

## DISCUSSION

Ply has multiple effects on bacterial/host interactions, and Ply‐deficient *S. pneumoniae* are less virulent in infection models [9, 10]. Although several publications have shown Ply is pro‐inflammatory [10, 13, 14, 15, 20, 22], more recent data demonstrated an anti‐inflammatory effect of Ply mediated by direct Ply binding to MRC‐1 expressed on human DCs and mouse macrophages [22]. The data presented here has extended this finding to a human macrophage population and used transcriptional analyses to characterize the effects in detail. Using multiple Ply‐deficient strains and an in vitro model of *S. pneumoniae* infection of human macrophages [7, 27] we have shown that Ply significantly impairs the MDM pro‐inflammatory transcriptional response to *S. pneumoniae*, resulting in major reductions in TNF production. Non‐haemolytic Ply suppressed MDM TNF responses to a lesser degree than haemolytic Ply, compatible with the known reduced level of binding to MRC‐1 for non‐haemolytic Ply [22]. We have also now shown Ply‐mediated suppression of MDM TNF responses was dependent on SOCS1. Overall, the data demonstrate that the dominant effect of Ply during the early interactions of macrophages with *S. pneumoniae* was to suppress the pro‐inflammatory cytokine response.

Systems level analysis revealed that rather than altering the qualitative nature of the response of the MDM response to *S. pneumoniae*, Ply attenuated the genome‐wide transcriptional response and reduced expression of multiple individual pro‐inflammatory genes. This resulted in a roughly twofold increase in TNF and several‐fold increase in IL‐6 release by MDMs after infection with TIGR4Δ*ply* compared to TIGR4. This suggests Ply reduces the signal strength of the inflammatory response, an effect supported by our data showing reduced NFκB activation in response to TIGR4 compared to TIGR4Δ*ply*, and the abrogation of differences in TNF production between these strains in the presence of a SOCS1 inhibitor. The transcriptional analysis also confirmed that Ply‐mediated suppression of pro‐inflammatory responses were not dependent on *S. pneumoniae* internalization by macrophages, compatible with being mediated by cell surface MRC‐1 rather than through leak of phagosome contents through Ply pores formed in the phagosome membrane.

Shenoy et al demonstrated that Ply‐mediated suppression of inflammatory responses was related to Ply‐dependent killing of macrophages [21]. In their data there was approximately 70% cell death of their mouse macrophage cell line when incubated with TIGR4 using a similar MOI and timepoint as our data. However, in our MDM model three separate cell death assays demonstrated low levels of MDM cell death which was not consistently higher after infection with TIGR4 compared to TIGR4Δ*pl*y. In addition, the transcriptome data were corrected for RNA quality and concentration thereby excluding cell death as an explanation for the genome‐wide effects we have described. Furthermore, microscopy showed increased NFκB activation in response to TIGR4Δ*pl*y compared to TIGR4 within 1 hr of infection in live MDMs. Hence differences in cell death are unlikely to explain the suppression of overall transcriptional response and the twofold decrease in the levels of TNF released by MDMs in response to TIGR4 compared to TIGR4Δ*pl*y. The difference in cell death between our data and Shenoy et al. may reflect variations in sensitivity of the cell types to the cytotoxic effects of Ply, or tissue‐dependent differences in Ply expression. We suggest both Ply‐mediated suppression of macrophage responses and cytotoxicity will contribute to varying degrees to the suppression of inflammatory responses to *S. pneumoniae* at different sites and intensity of infection. We have previously shown human MDMs and alveolar macrophages have similar transcriptional responses to stimulation with lipopolysaccharide [37], and our data and Subramanian et al. [22] confirm that the effects of Ply seen in vitro are also relevant during infection with increased BALF TNF levels after infection with TIGR4Δ*ply* compared to TIGR4 despite having lower BALF CFU levels.

Although our results seemingly contradict previous papers showing Ply has pro‐inflammatory effects, both anti‐ and pro‐inflammatory effects of Ply could co‐exist. Indeed, during early lung infection with TIGR4 despite reduced BALF TNF levels, the levels of IL‐1β were increased compared to infection with TIGR4Δ*ply*. Potentially Ply‐mediated suppression of pro‐inflammatory gene expression was counterbalanced by Ply‐dependent activation of the inflammasome. Overall, the effects of Ply on host inflammatory responses is likely to be a dynamic process that evolves over time with variable effects on individual cytokines depending on whether they are inflammasome‐dependent or ‐independent.

In summary we have shown the early pro‐inflammatory macrophage response to *S. pneumoniae* in vitro and in a mouse infection model were inhibited by Ply. This effect was mediated through SOCS1, and rather than a qualitative change in the inflammatory response resulted in a general reduction in expression of multiple pro‐inflammatory genes including TNF and IL‐6.

## AUTHOR CONTRIBUTIONS

Jimstan Periselneris conducted experiments, analysed data, and cowrote the manuscript. Carolin T. Turner analysed data. Gabriella Szylar conducted experiments. Giuseppe Ercoli conducted experiments and analysed data. Caroline M. Weight conducted experiments and analysed data. Teresa Thurston analysed data. Gillian Tomlinson conducted experiments. Mahdad Noursadeghi analysed data and cowrote the manuscript. Jeremy Brown analysed data and cowrote the manuscript. All authors approved the final draft of the manuscript.

## CONFLICT OF INTEREST

The authors declare no conflict of interest.

## Supporting information


**Figure S1** Measurement of Ply cytotoxicity activity using the red cell lysis assay. Ply activity in vitro for different *S. pneumoniae* (A) TIGR4 or (B) D39 strains. Haemolysis was measured by microplate spectrophotometer at 540 nm with high absorbance representing more haemolysis. 0.5% Saponin was used as a positive control.

**Figure S2** Effects of Ply on MDM cell death assessed with a tetrazolium based cell viability kit and TNF production measured using ELISA. (A) MDM viability after incubation with TIGR4 or TIGR4Δ*ply* at MOI 10 for 4 and 6 hours. (B to D) Data for MDMs with and without treatment with the pan caspase inhibitor 20 μM ZVAD FMK (Invivogen) or the caspase 1 inhibitor 50 μM YVAD FMK (Invivogen). (B) Cell viability results. Data are presented as means +/− SEM and analysed by 2 way ANOVA with Sidak multiple comparisons test. (C and D) Supernatant TNF levels, with data presented as means +/− SEM of 3 experiments and analysed by 1 way ANOVA with Tukey's multiple comparisons test. For panel A, no statistically significant differences were observed for result for TIGR4 compared to TIGR4Δ*ply*. For panels B to D, no statistically significant differences were observed for result with or without addition of ZVAD or YVAD for an individual strain.

**Figure S3** Correlation of mean fold change in expression of individual genes from transcriptome of MDMs incubated with TIGR4 (MOI 10) versus unstimulated MDM (for 4 hours) compared to MDMs infected with TIGR4Δ*ply* versus unstimulated MDM with (A) and without (B) addition of cytochalasin D to inhibit phagocytosis. Data were derived from 3 separate experiments for each condition.

## Data Availability

The data that support the findings of this study are openly available in ArrayExpress (https://www.ebi.ac.uk/arrayexpress/, accession number: E‐MTAB‐8947).
