# Supplementary figures and images for: Pneumolysin suppresses the initial macrophage pro‐inflammatory response to Streptococcus pneumoniae
Source: Immunology. 2022 Jul 28;167(3):413–27. doi: 10.1111/imm.13546 (PMC10497322; doi:10.1111/imm.13546)

A

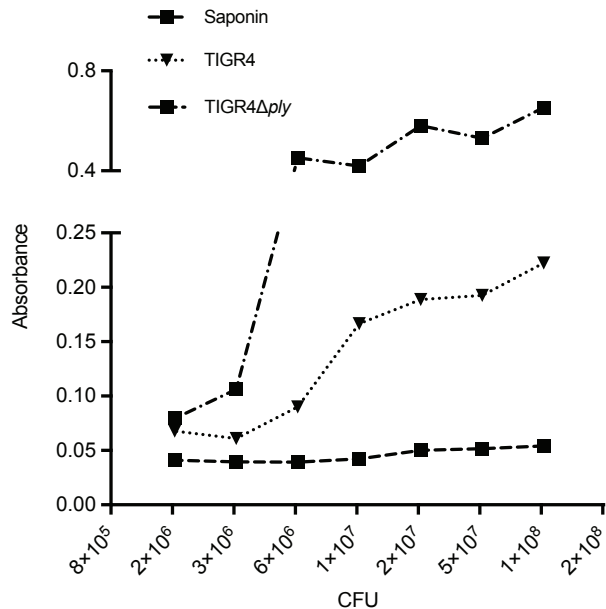

B

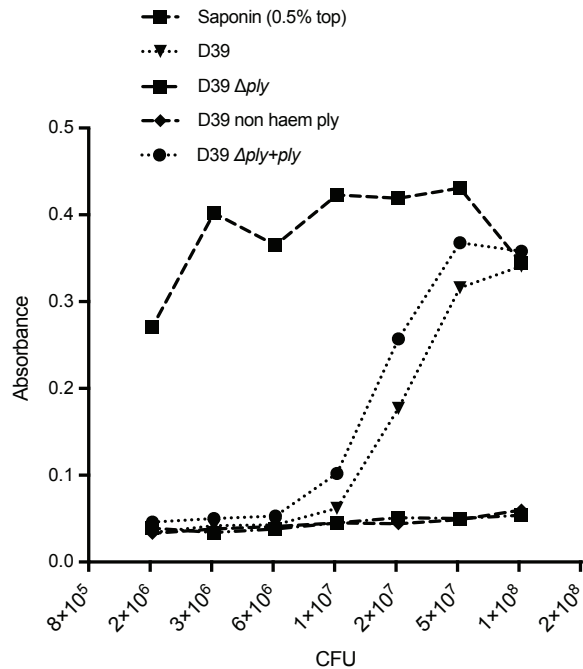

Supplement: Supplementary file 1 — Figure S1 Measurement of Ply cytotoxicity activity using the red cell lysis assay. Ply activity in vitro for different S. pneumoniae (A) TIGR4 or (B) D39 strains. Haemolysis was measured by microplate spectrophotometer at 540 nm with high absorbance representing more haemolysis. 0.5% Saponin was used as a positive control. [file IMM-167-413-s003.pdf]

**A**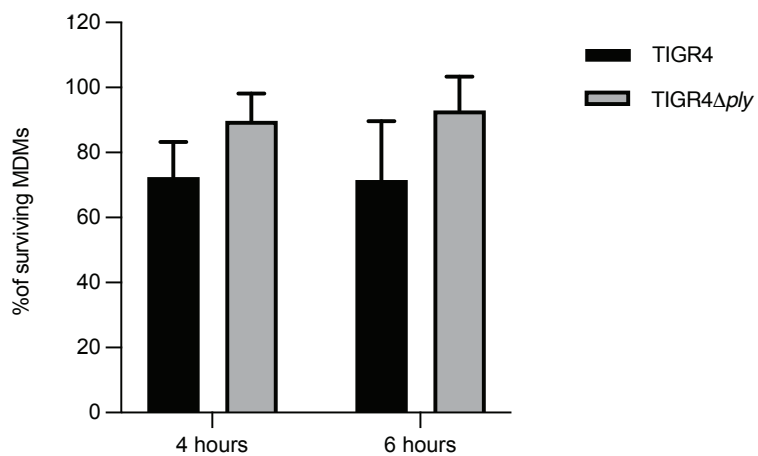**B**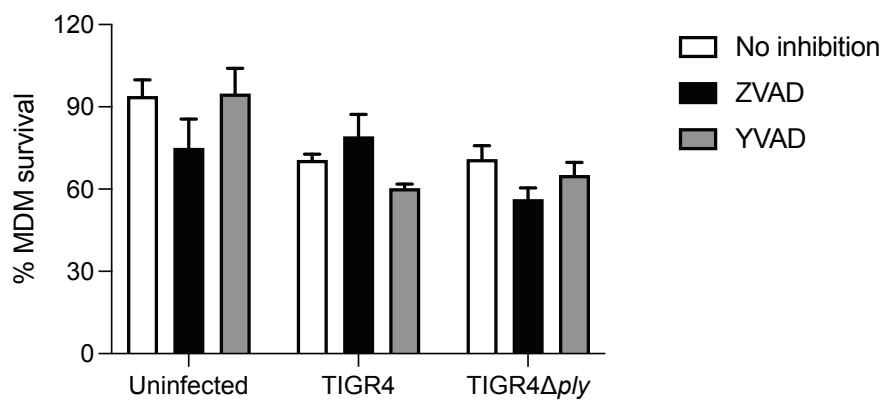**C**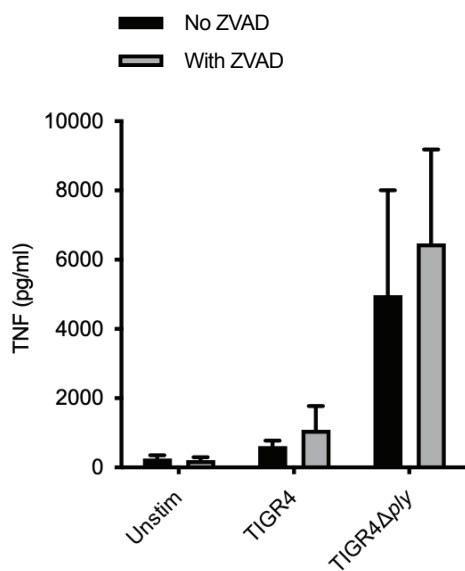**D**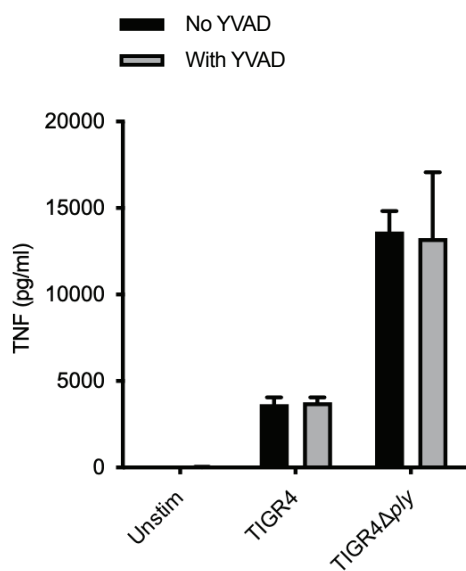

Supplement: Supplementary file 2 — Figure S2 Effects of Ply on MDM cell death assessed with a tetrazolium based cell viability kit and TNF production measured using ELISA. (A) MDM viability after incubation with TIGR4 or TIGR4Δply at MOI 10 for 4 and 6 hours. (B to D) Data for MDMs with and without treatment with the pan caspase inhibitor 20 μM ZVAD FMK (Invivogen) or the caspase 1 inhibitor 50 μM YVAD FMK (Invivogen). (B) Cell viability results. Data are presented as means +/− SEM and analysed by 2 way ANOVA with Sidak multiple comparisons test. (C and D) Supernatant TNF levels, with data presented as means +/− SEM of 3 experiments and analysed by 1 way ANOVA with Tukey's multiple comparisons test. For panel A, no statistically significant differences were observed for result for TIGR4 compared to TIGR4Δply. For panels B to D, no statistically significant differences were observed for result with or without addition of ZVAD or YVAD for an individual strain. [file IMM-167-413-s001.pdf]

**A**

No cytochalasin D

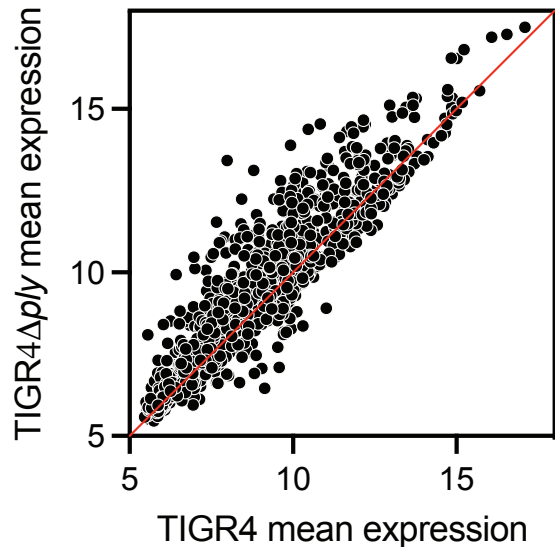**B**

With cytochalasin D

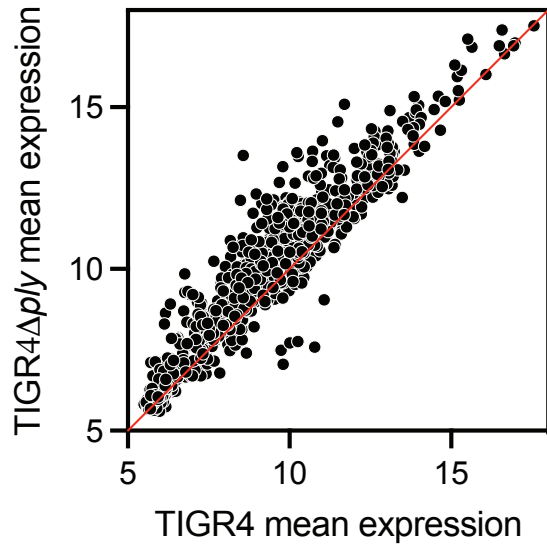

Supplement: Supplementary file 3 — Figure S3 Correlation of mean fold change in expression of individual genes from transcriptome of MDMs incubated with TIGR4 (MOI 10) versus unstimulated MDM (for 4 hours) compared to MDMs infected with TIGR4Δply versus unstimulated MDM with (A) and without (B) addition of cytochalasin D to inhibit phagocytosis. Data were derived from 3 separate experiments for each condition. [file IMM-167-413-s002.pdf]
